# Zinc/CaMK II Associated-Mitophagy Signaling Contributed to Hippocampal Mossy Fiber Sprouting and Cognitive Deficits Following Neonatal Seizures and Its Regulation by Chronic Leptin Treatment

**DOI:** 10.3389/fneur.2018.00802

**Published:** 2018-09-26

**Authors:** Li-li Li, Mei-fang Jin, Hong Ni

**Affiliations:** Neurology Laboratory, Institute of Pediatric Research, Children's Hospital of Soochow University, Suzhou, China

**Keywords:** leptin, zinc transporter, mossy fiber spouting, mitotophagy, CaMK II

## Abstract

The role of leptin in the pathogenesis of epilepsy is getting more and more attention in clinical and basic research. Although there are data indicating neuroprotective effects of elevated serum/brain leptin levels following acute seizures, no study to date has dealt with the impact of chronic leptin treatment on long-term brain injury following developmental seizures. The aim of this study was to evaluate whether chronic leptin treatment may have neuroprotective effects on cognitive and hippocampal mossy fiber sprouting following flurothyl-induced recurrent neonatal seizures and whether these effects are mediated by the zinc/CaMKII-associated mitophagy signaling pathway. Forty Sprague-Dawley rats (postnatal day 6, P6) were randomly assigned into two groups: neonatal seizure group and control group. At P13, they were further divided into control group, seizure group (RS), control + leptin (leptin, i.p., 2 mg/kg/day for 10 days), seizure+leptin group (RS+Leptin, 2mg/kg/day, i.p., for 10 consecutive days). Morris water maze test was performed during P27-P32. Subsequently, Timm staining and Western blotting were used to detect the mossy fiber sprouting and protein levels in hippocampus. Flurothyl-induced seizures (RS group) significantly down-regulated mitophagy markers PINK, Drp1, PHB, and memory marker CaMK II alpha while up-regulating zinc transporters ZnT3, ZnT4, ZIP7, and autophagy execution molecular cathepsin-E, which were paralleled with hippocampal aberrant mossy fiber sprouting and cognitive dysfunction. However, these changes were restored by chronic leptin treatment (RS+Leptin group). The results showed that leptin had neuroprotective effect on hippocampal pathological damage and cognitive deficits induced by neonatal seizures and suggested that Zinc/CaMK II associated-mitophagy signaling pathway in hippocampus may be a new target of leptin's neuroprotection, with potential value of translational medicine.

## Introduction

Leptin, cloned in 1994 from white adipocytes, functions initially as an ob gene to reduce obesity by controlling appetitive behaviors via hypothalamic neurons (1). In the past decade, however, leptin receptors have widely been discovered in diverse brain regions, especially hippocampus, which plays a role in hippocampal-dependent neuronal morphology, synaptic plasticity, and cognition (2, 3). Patients with leptin deficiency due to mutations in the ob gene showed structural and functional changes in the brain, including the hippocampus, and replacement therapy with leptin corrects for these changes (4). It is noteworthy that leptin is often located at axonal and synaptic subcellular sites (5), suggesting that it regulates neuronal excitation conduction and synaptic currents. In consistent, leptin inhibits epileptiform-like activity in hippocampal neurons (6) and suppresses seizures in two rodent seizure models (7, 8). Leptin deficient ob/ob mice exhibited an increased severity of pentylenetetrazol-induced seizures (9). Similarly, the neuroprotective effect of leptin in the status epilepticus induced by kainic acid or pilocarpine was also reported (10, 11).

Among the molecular mechanisms responsible for the effects of leptin, the Ca(2+)/Zn(2+)-permeable AMPA (α-amino-3-hydroxy-5-methyl-4- isoxazolepropionic acid, AMPA)-mediated PI3K activation appears to be a target of leptin involved in hippocampal-dependent cognition, neuronal morphology and activity-dependent synaptic plasticity (12). Activation of synaptic GluR2-deficient AMPA receptor insertion mediated leptin-induced suppression of excitability of hippocampal neurons (13, 14). Leptin attenuated cerebral ischemia injury by promote the energy metabolism via PI3K/Akt (15). As it has been documented that reducing the expression of Zn(2+)-impermeable GluR2(R) channels rescues vulnerable CA1 pyramidal neurons from forebrain ischemic injury (16), and that PI3K signaling is a key upstream regulator of autophagy that contributes to mTOR activation (17), thus, it is reasonable to speculate that zinc/autophagic signaling may be involved in the above mentioned effects of leptin.

More recently, we developed a “twice hit” epileptic seizure model that initially used pilocarpine to induce status epilepticus (SE) in neonate and then used penicillin to induce a second SE in adolescence or adulthood. The results showed that leptin treatment can prevent long-term decline in seizure threshold caused by neonatal seizures and the abnormal expression of hippocampal mossy fiber sprouting-related ZnT3/CB-D28k (18). Our previous studies demonstrated that seizures during developmental period lead to long-term up-regulation of zinc ion transporters ZnT-3, MT-3, lipid transporters ApoE and clusterin, and ACAT-1 (a catalytic esterification of cholesterol and long chain fatty acids to form cholesterol Ester mitochondrial localization enzyme) in hippocampus, accompanied by abnormal mossy fiber sprouting and cognitive impairment. KD treatment restored these changes. The results suggested that zinc-induced lipid peroxidation and mitochondrial metabolic pathway may be the therapeutic target of KD (19). Chronic KD treatment restored the up-regulated clusterin and autophagy-related genes Beclin-1, p62 and Cathepsin-E following neonatal seizure, indicating that the autophagy signaling may be involved in the zinc/mitochondrial metabolic pathway (20).

Despite these results, direct evidence for zinc/mitochondrial associated mitophagy signaling to be involved in the action of this adipokine during neuroprotection following developmental seizures is lacking. Therefore, in the present study, the effects of leptin treatment on cognition and hippocampal mossy fiber sprouting, as well as related gene expressions, including zinc transporters (ZnT-3, ZnT-4, ZIP7), the mitophagy signaling molecular (PHB, PINK1, Drp1, Cathepsin-E) and the memory molecular CaMK IIα, were examined following flurothyl-induced recurrent neonatal seizures.

## Materials and methods

### Animal preparation

Forty Sprague-Dawley rats on postnatal day 6 (P6) were obtained from the Chinese Academy of Sciences, Shanghai Experimental Animal Center, China. The animals were treated in accordance with the guidelines set by the National Institutes of Health for the humane treatment of animals. Take adequate measures to minimize the amount of pain and animals. Animals were randomly assigned to two groups: the flurothyl (bis-2, 2, 2-triflurothyl ether; Sigma-Aldrich Chemical, WI, USA)-induced recurrent seizures group (*n* = 20) and the control group (*n* = 20). From P6 to P12, seizures were induced with flurothyl for 30 min per day for consecutive 7 days in recurrent seizures group (20), with a flow rate of 100 μl/min onto filter paper in the center of the container where it evaporated (21). Flurothyl (bis-2,2,2-triflurothyl ether) is an inhaled anesthetic that induces generalized tonic-clonic seizures in the central nervous system, which is effective and rapid acting. Animals will lose consciousness when they have just inhaled trifluoroethyl ether, and continued inhalation will produce epileptic seizure-like seizures (Since the animal was just born and the age is too small, it is not possible to record EEG at this stage). The seizure induced by this method is very simple and the effectiveness is comparable to the electric shock method. Moreover, the action of trifluoroethyl ether mainly inhibits the descending central nervous system, has no inhibitory effect on the medulla oblongata, and can effectively prevent animal death caused by respiratory depression when induced seizures. After 40–60 s, the seizures occurred. The rats showed dysphoria, head nodding, jumping, accompanied by screams, followed by skin mucous cyanosis, limb stiffness and paralysis, namely generalized tonic-clonic seizure and other seizure behaviors according to Racine scores at level III or above (Racine grade III: acial clonus, including spasm-like blink, beard moving, rhythmic mastication, rhythmic nod, forelimb clonus). Each episode lasted 4–5 min, then followed by intermittence for 2 to 3 min, meanwhile the consciousness was not restored. The rats in the control group were normal and had no seizures. At P13, all rats were further divided into the control group without leptin (Control, *n* = 10), the control group plus i.p. leptin treatment (Leptin, *n* = 10), the recurrent seizures group without leptin treatment (RS, *n* = 10) and the RS plus i.p. leptin treatment group (RS+Leptin, *n* = 10). Murine leptin was obtained lyophilized from Peprotech Inc. (Rocky Hill, NJ) and reconstituted in 0.01 M PBS buffer. From P13 to P22, the animals in the Leptin group and the RS+Leptin group were given leptin (2 mg/kg, 1 ml/kg, i.p.). Control rats were injected with the same volume (1 ml/kg) of vehicle (0.01 M PBS). The four groups of animals were in the same feeding conditions.

### Open field test

The open field test was used to assess the movement and exploration activities of rats on P33 (22). The device was washed with a 5% ethanol-water solution prior to placing the animals to avoid deviations caused by odor cues left by previous animals. Animals were tested in a 30 cm walled square acrylic arena (72 × 72 cm). The floor of the arena was divided into 16 squares of 18 × 18 cm, and the mice were placed in the center of the arena and observed for 5 min. The following parameters were measured: 1) total locomotion (total number of lines crossed with four paws); 2) grooming (the number of times the body was cleaned with the claws, combing the body and pubis with the mouth, washing the face), and 3) feeding (the rodent stood in the hind legs) The number of times. 1) crossing (the four claws); 2) grooming (the number of times the body was cleaned with the claws, combing the body and pubis with the mouth, washing the face), and 3) rearing (number of times the rodents stood on their hind legs).

### Morris water maze test(MWM)

To assess visual spatial learning and memory abilities, five rats were randomly selected in each group for MWM test on P28 to P32 (*n* = 5/group) according to the method described previously (23). In short, for place navigation tests, the escape latency was automatically recorded using a video/computer system (the duration of each rat seeking a platform in the water maze). For the space probe test, the platform was removed from the pool the day after the navigation test (P33). Each rat was placed in water for 60 s and the number of passes through the platform quadrant was recorded.

### Timm staining

On P34, the day after the water maze test, five rats were randomly selected in each group and given chloral hydrate (injection dose of 1 ml/100 g). After the anesthesia, the rats in each group were perfused through cardioplegia in the order of 0.9% normal saline (80 ml), 4% paraformaldehyde PB solution (100 ml), 0.4% sodium sulfide (80 ml) and 4% paraformaldehyde PB solution (60 ml). The method of Timm staining (*n* = 5/each group, P34) has been described previously (20). Arabic gum powder (45 g) was dissolved in 90 ml of double distilled water. After standing at room temperature for 48 h, the gum arabic buffer was mixed with citrate buffer (15 ml), 5.67% hydroquinone (45 ml) and 17% silver nitrate (0.5 ml) in the dark, and the mixture was poured into the dyeing tank containing the slices. The sections were stained in the dark in a 37°C water bath for about 1–2 h. The sections were rinsed in running water for about 30 min, dehydrated and dried with a conventional gradient alcohol and sealed with a nresin. Semi-quantitative evaluation of mossy fiber sprouting was recorded by recording the timm score in dentate gyrus(DG)and CA3 region. Sections from each animal were assigned a score from 0 to 3 related to the quantity of supragranular mossy fiber-like staining. Sections with no or only occasional supragranular mossy fiber-like staining were scored 0. Sections with scattered mossy fiber-like staining above all parts of the granular layer were given a score of 1. Sections that exhibited either patches of heavy mossy fiber-like staining interspersed with regions of sparser staining or a continuous band of staining intermediate in intensity between sections scored 1 and 3 were given a score of 2. Finally, sections with a dense, continuous band of supragranular mossy fiber-like staining were assigned a score of 3 (24). The person evaluating timm score was blind to the grouping.

### Western blot analysis

Five rats in each group were randomly selected and sacrificed after intraperitoneal injection of 4% chloral hydrate (1 ml/100 g). The isolated hippocampus tissues were quickly transferred to pre-labeled EP tubes precooled on dry ice. The Western blot method has been described previously (*n* = 5/group, P34) (25). Briefly, after blocking, the polyvinylidene fluoride membrane blots were incubated with one of the following antibodies: goat anti-ZnT3 polyclonal antibody (1:100, Santa Cruz), goat anti-ZnT4 polyclonal antibody (1:100, Santa Cruz), goat anti-ZIP7 polyclonal antibody (1:100, Santa Cruz), rabbit anti-PHB polyclonal antibody (1:2000, Abcam), rabbit anti-PINK1 polyclonal antibody (1:1000, Abcam), mouse anti-DRP1 monoclonal antibody (1:500, Abcam), rabbit anti-Cathepsin E polyclonal antibody (1:1000, Abcam), rabbit anti-CaMKII_α_ polyclonal antibody (1:2000, Abcam), rabbit anti-Leptin polyclonal antibody (1:2000, Abcam) or rabbit anti-GAPDH polyclonal antibody (1:5000, Beyotime Biotechnology) in TBST contain 5% nonfat dry milk overnight at 4°C. After washing with TBST 3 times, the blot was incubated with the secondary antibody for approximately 2 h at ambient temperature. The antibody reactions were exposed with Kodak X-ray film using the ECL assay system, Amersham imager 600 (GE Healthcare, USA). The intensity of each band was analyzed by Image Pro Plus (IPP) software.

### Co-immunoprecipitation (co-IP)

Co-immunoprecipitation (co-IP) assay was performed according to the Santa Cruz immunoprecipitation protocol. Extracts of hippocampus were lysed with RIPA lysis buffer (Beyotime Biotechnology). The supernatants were incubated with ZnT4 antibody(1ug) or goat IgG(1ug) for 2 h at 4 °C. Protein A / G agarose (30 ul, Santa Cruz) was added and the mixtures were incubated overnight at 4° C. Agarose containing protein complexes were washed 5 times with lysis buffer. After the last wash, the supernatant is aspirated and discarded and the pellet resuspended in 50 μL of lysis buffer and 5 × loading buffer. Boil the sample for 5 min. Continue with electrophoresis and immunoblotting as described under Western blotting procedure with goat anti- ZnT4 and mouse anti-DRP1 antibodies.

### Statistical analysis

Escape latency was analyzed using two-way, repeated-measures ANOVAs (treatment as a between subject factor and training day as a within subject factor). The frequency of passing through the platform quadrant of spatial probe test in the water maze, Timm staining and the protein levels were analyzed with one-factor ANOVA with *post hocs*. Correlation analysis was performed with the Pearson method. Data were presented as the mean ± SD. Statistical significance was considered a *P* < 0.05.

## Results

### Open field test

**Figure 1:**
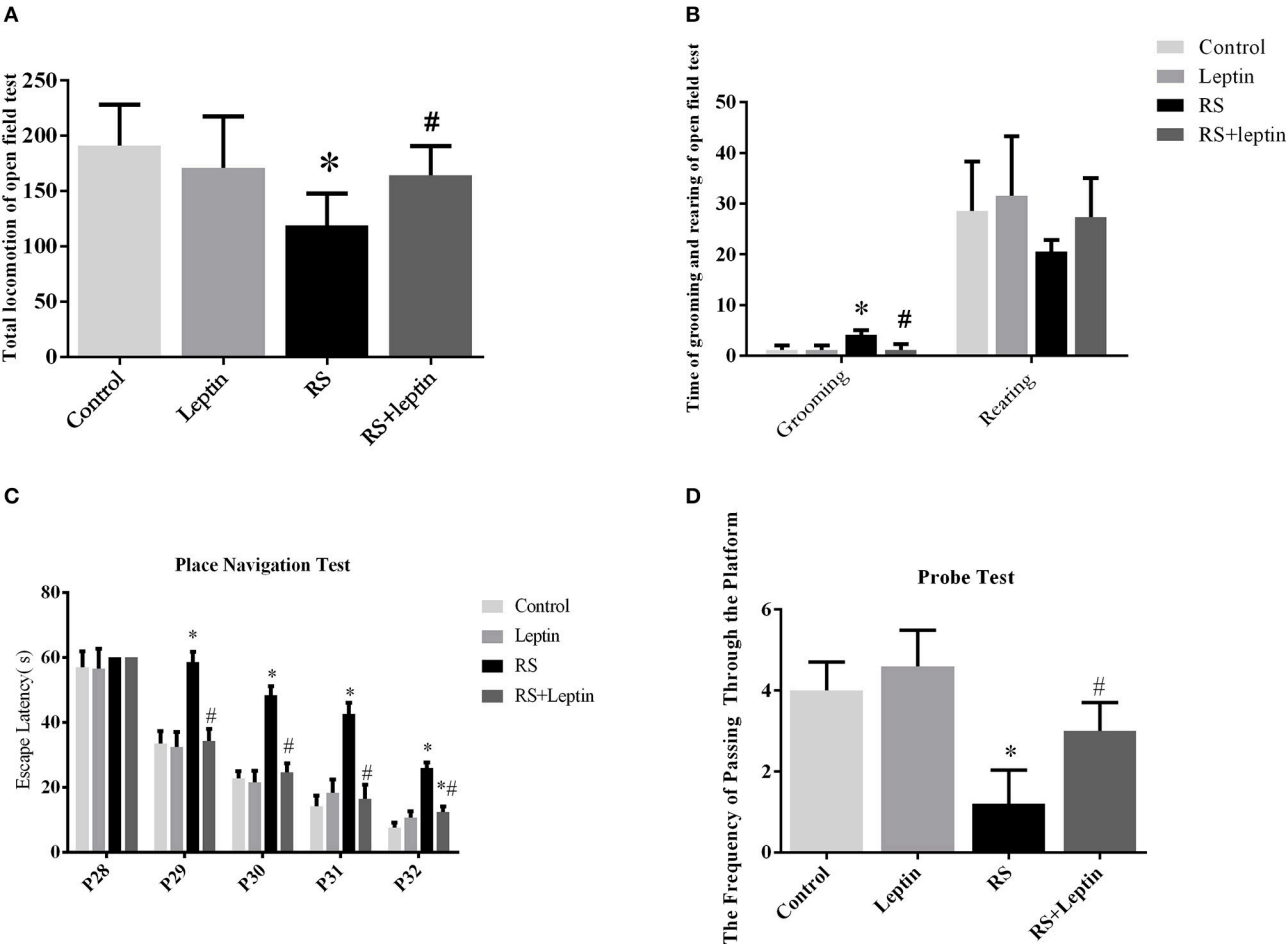
The open field test. **(A)** Total locomotion in the open field test on P33. **(B)** Number of grooming and rearing times in the open field test on P33.The Morris water maze test. **(C)** During the 5 days of training, the escape latency for each group in the place navigation test was plotted. **(D)** The frequencies of crossing the platform in the probe test on P33. ^*^P < 0.05, compared with control, ^#^*P* < 0.05, compared with RS (n = 5/group).

The open field test was used to test the spatial exploration behavior of rodents (Figures 1A,B). The motor activity scores of the RS group were significantly lower than those of the control group and the leptin group (*P* = 3.67, *P* < 0.05). The motor activity scores of the leptin-treated RS+leptin group were significantly increased compared to the RS group (Figure 1A). On P33, the RS group showed significantly more grooming than the control group (F = 13.64, *P* < 0.0001). The grooming time of the leptin-treated RS+leptin group was significantly reduced compared to the RS group (Figure 1B). The RS group showed less rearing activity than the control group, but was not statistically significant (Figure 1B).

### MWM

The escape delay data for each group is shown in Figure 1C. During the 5 days of training, the escape latency for all animals were gradually decreased. On days 2–5 of training (P29-P32), the RS group took more time to find the platform than the control group and the RS + leptin group (*P* < 0.05). A two-way repeated ANOVA of escape latencies for the training days revealed significant group differences [*F*_(3, 12)_ = 92.07, *P* < 0.0001] and training day effect [*F*_(4, 16)_ = 976.2, *P* < 0.0001], as well as significant interaction of group and day [*F*_(12, 48)_ = 17.52, *P* < 0.0001]. *Post hoc* comparison showed there was significant difference between RS group and RS + leptin group on the 2nd to 5th days of training, and the difference between RS group and control group was also statistically significant (*P* < 0.05). In the space probe test, the frequency of crossing quadrant of RS group was significantly lower than that of control group, and leptin injection could reverse this situation (*P* < 0.05; Figure 1D).

### Timm staining

**Figure 2:**
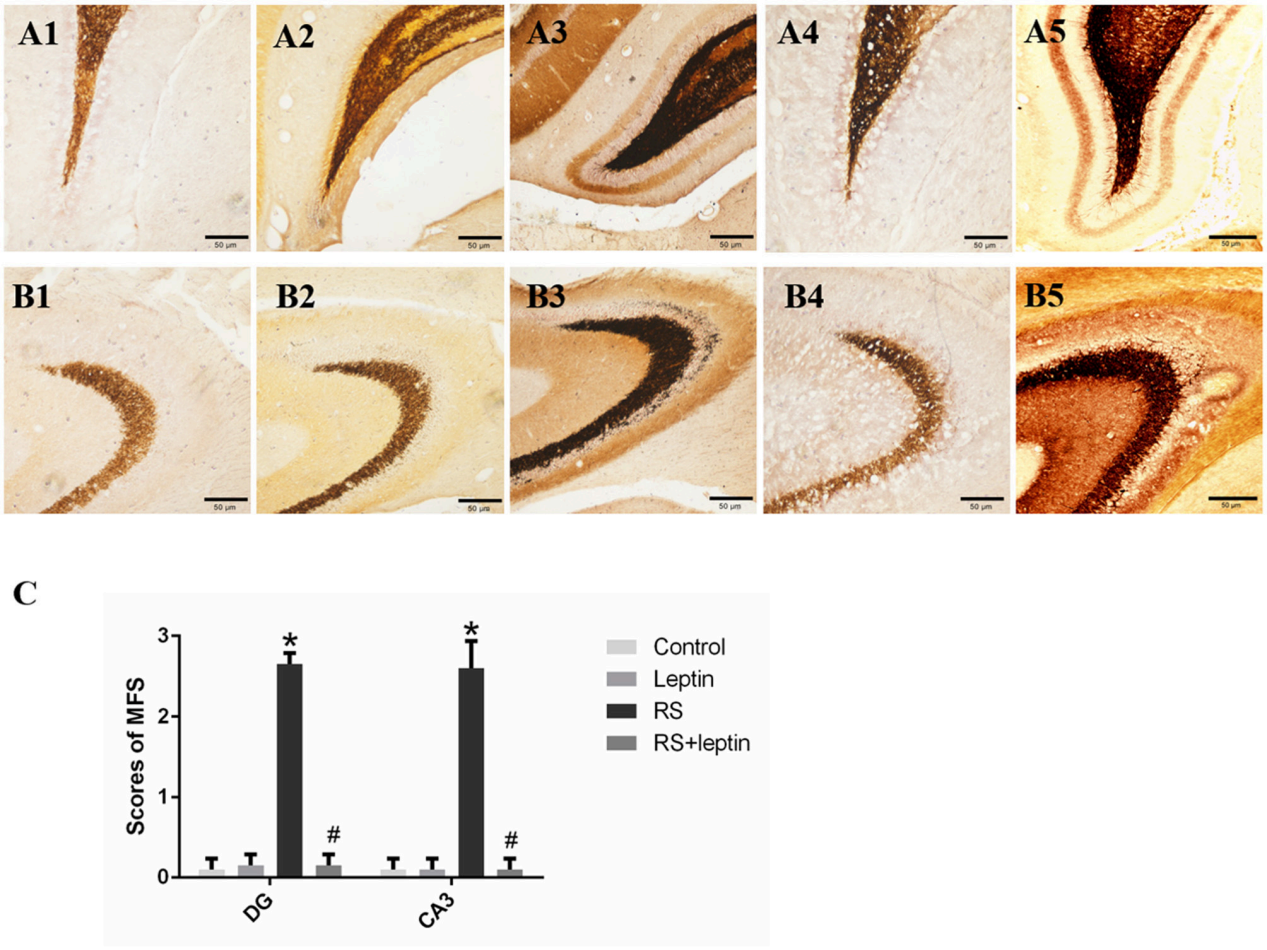
**(A,B)** Mossy fiber sprouting by Timm staining in control **(A1, B1)**, leptin **(A2, B2)**, RS **(A3, B3)** and RS +leptin **(A4, B4)**. **A1–A4** represent dentate gyrus subfield and B1-B4 represent CA3 subfield from control to RS+ leptin respectively. Note the excessive amount of Timm staining in the inner molecular layer of the granule cells **(A3)** and the stratum pyramidale of CA3 subfield **(B3)** in RS group (arrows). Calibration bars = 50 μm (*n* = 5/group). **(A5**) Positive control of DG subfield for the Timm (pilocarpine-induced SE), **(B5)** Positive control of CA3 subfield for the Timm (pilocarpine-induced SE). **(C)** Timm score of dentate gyrus subfield and CA3 subfield from control to RS+ leptin. ^*^P < 0.05, compared with control, ^#^*P* < 0.05, compared with RS (*n* = 5/group).

As shown in Figure 2, the RS group has significant Timm particles on the supragranular of DG (Figure 2A3) and CA3 subfield (Figure 2B3). However, Timm particles were barely visible in the control (Figures 2A1, B1) and leptin (Figures 2A2, B2) and RS + leptin groups (Figures 2A4, B4). Timm score analysis showed that Timm score in RS group was significantly higher than that in control group (*P* < 0.05; Figure 2C). In addition, RS + leptin group scores were significantly lower than the RS group (CA3: F = 185.2, *P* < 0.0001; DG: F = 422.4, *P* < 0.0001).

### Western blot

**Figure 3:**
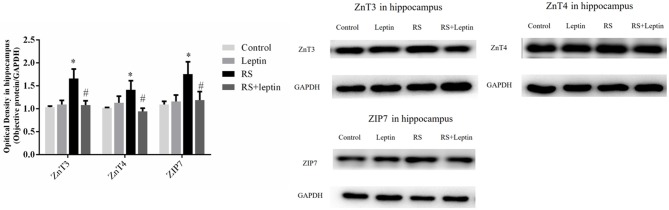
Protein levels of ZnT3, ZnT4, and ZIP7 in hippocampus. Extracts of hippocampus from the four groups were separated on SDS-PAGE, and protein levels were detected by Western blot method. ^*^P < 0.05, compared with control, ^#^*P* < 0.05, compared with RS (*n* = 5/group).

**Figure 4:**
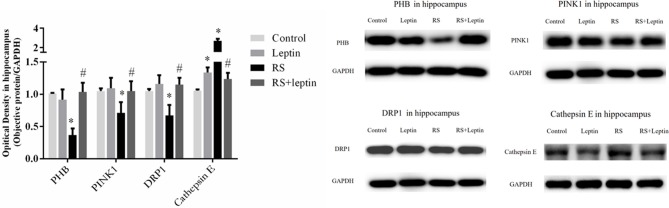
Protein levels of PHB, PINK1, DRP1 and Cathepsin E in hippocampus. Extracts of hippocampus from the four groups were separated on SDS-PAGE, and protein levels were detected by Western blot method. ^*^P < 0.05, compared with control, ^#^*P* < 0.05, compared with RS (*n* = 5/group).

**Figure 5:**
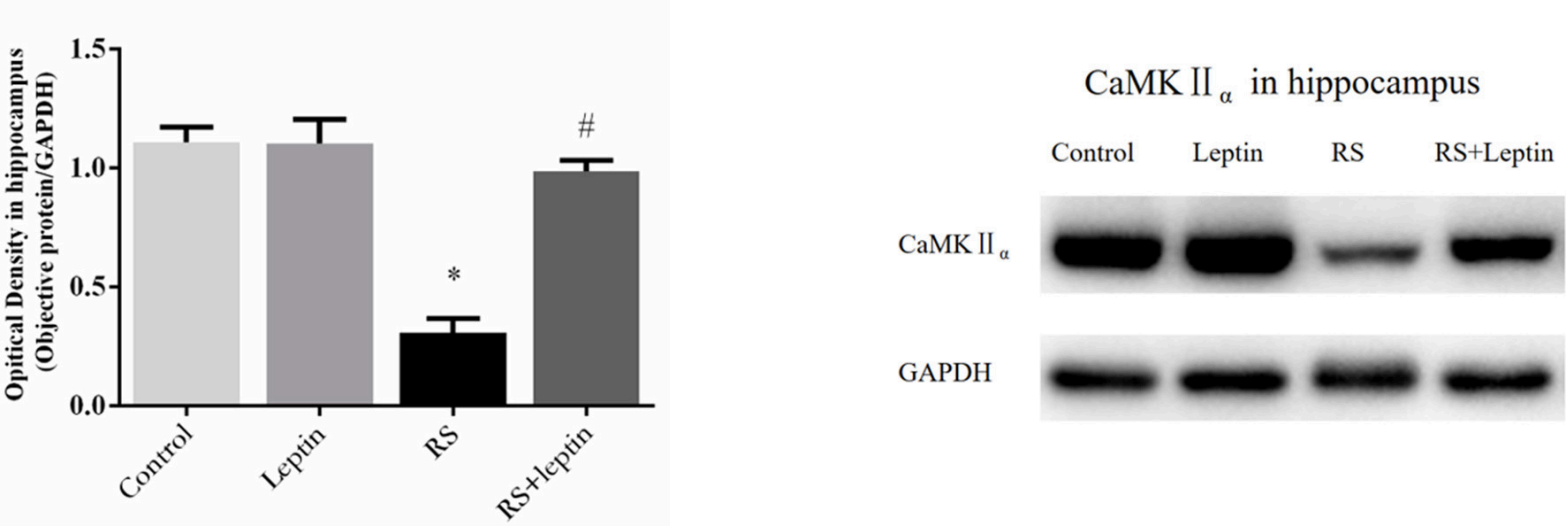
Protein levels of CaMKIIα in hippocampus. Extracts of hippocampus from the four groups were separated on SDS-PAGE, and protein levels were detected with Western blot analysis. ^*^P < 0.05, compared with control, ^#^*P* < 0.05, compared with RS (*n* = 5/group).

**Figure 6:**
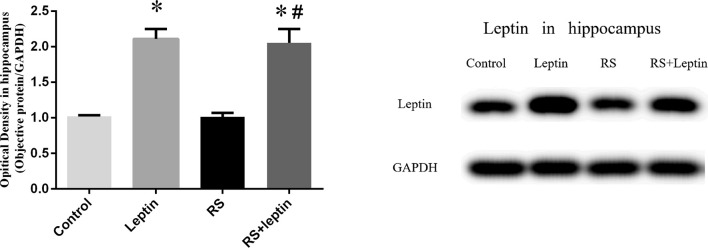
Protein levels of Leptin in hippocampus. Extracts of hippocampus from the four groups were separated on SDS-PAGE, and protein levels were detected with Western blot analysis. ^*^P < 0.05, compared with control, ^#^*P* < 0.05, compared with RS (*n* = 5/group).

Western blot was employed to evaluate the relative protein levels ZnT3, ZnT4, ZIP7, PHB, PINK1, DRP1, Cathepsin E, CaMKII_α_, and Leptin in hippocampus after Morris water maze analysis. As shown in Figures 3–5, there were down-regulated protein levels of PHB, PINK1, DRP1, CaMKII_α_ and up-regulated protein levels of ZnT3, ZnT4, ZIP7, and Cathepsin E in RS rats when compared with control rats(ZnT3: F = 44.47, *P* = 0.0002; ZnT4: F = 15.52, *P* < 0.05;ZIP7: F = 14.48, *P* < 0.0001; PHB: F = 35.46, *P* < 0.0001; PINK1: F = 7.82, *P* = 0.002; DRP1: F = 18.71, *P* < 0.0001; Cathepsin E: F = 186.50, *P* < 0.0001; CaMKII_α_: F = 144.00, *P* < 0.0001); these altered protein levels were reversed by chronic leptin treatment (*P* < 0.05). As shown in Figure 6, there were up-regulated protein levels of Leptin in leptin and RS+leptin rats[*F*_(3, 16)_ = 126.1,*P* < 0.0001].

### Correlation analysis

**Figure 7:**
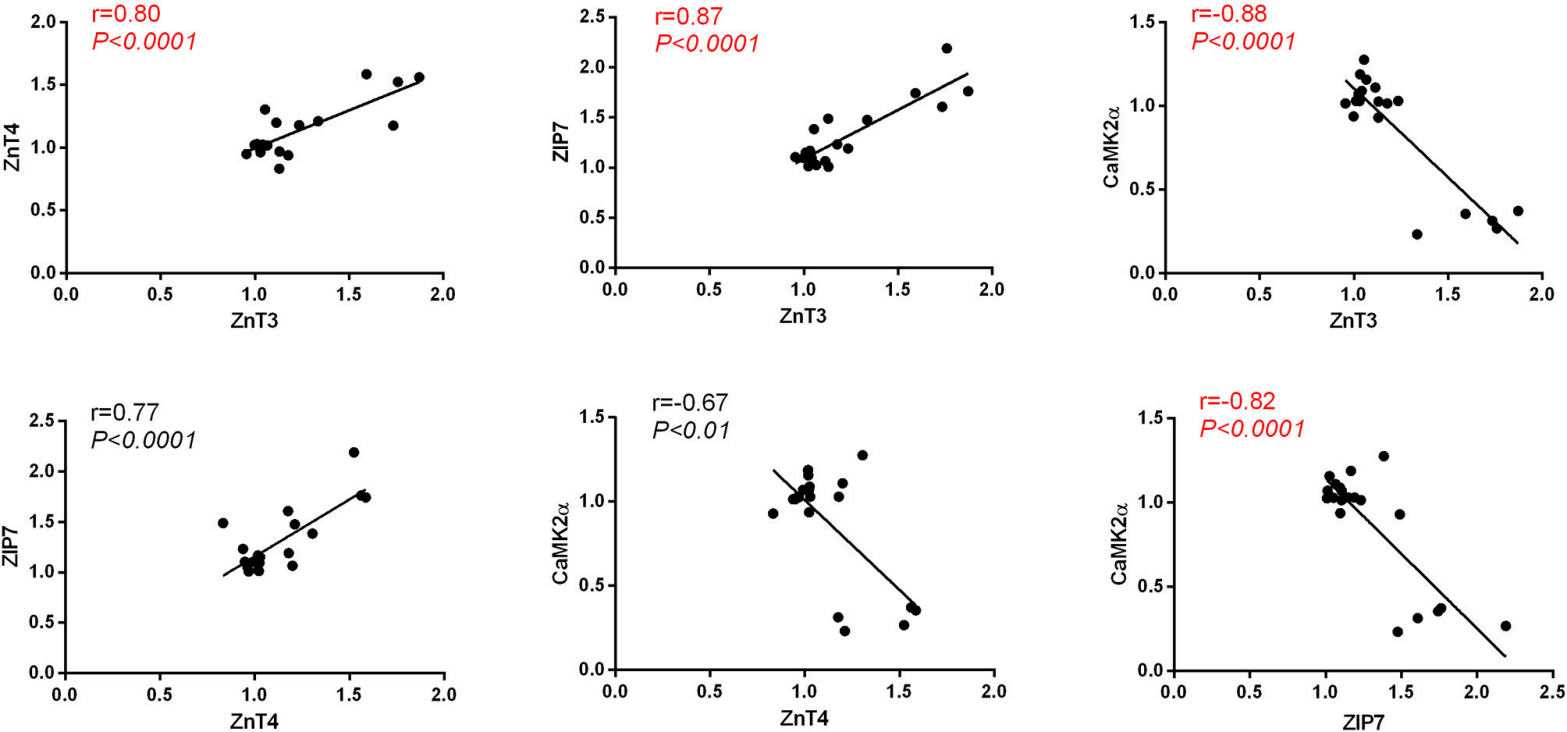
Pearson's correlation between the protein level of CaMKIIα, ZnT3, ZnT4 and ZIP7 in hippocampus.

We observed a strong positive correlation between the protein level of ZnT3 and ZnT4(Pearson's *r* = 0.80, *P* < 0.0001), ZnT3 and ZIP7(Pearson's *r* = 0.87, *P* < 0.0001), ZnT4 and ZIP7(Pearson's *r* = 0.77, *P* < 0.0001) and a strong negative correlation between the protein level of CaMKII_α_ and the levels of ZnT3(Pearson's *r* = −0.88, *P* < 0.0001), ZnT4 (Pearson's *r* = −0.67, *P* < 0.01) and ZIP7(Pearson's *r* = −0.82, *P* < 0.0001) in hippocampus (Figure 7).

**Figure 8:**
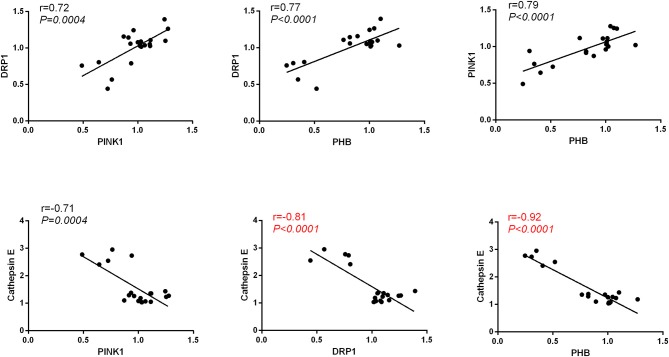
Pearson's correlation between the protein level of PHB, Pink1, DRP1, and Cathepsin E in hippocampus.

We observed positive correlation between the protein level of PHB and Pink1(Pearson's *r* = 0.79, *P* < 0.0001), PHB and DRP1(Pearson's *r* = 0.77, *P* < 0.0001), DRP1 and Pink1(Pearson's *r* = 0.72, *P* = 0.0004) and a strong negative correlation between the protein level of Cathepsin E and the levels of Pink1(Pearson's *r* = −0.71, *P* = 0.0004), DRP1(Pearson's *r* = −0.81, *P* < 0.0001) and PHB (Pearson's *r* = −0.92, *P* < 0.0001) in hippocampus (Figure 8).

**Figure 9:**
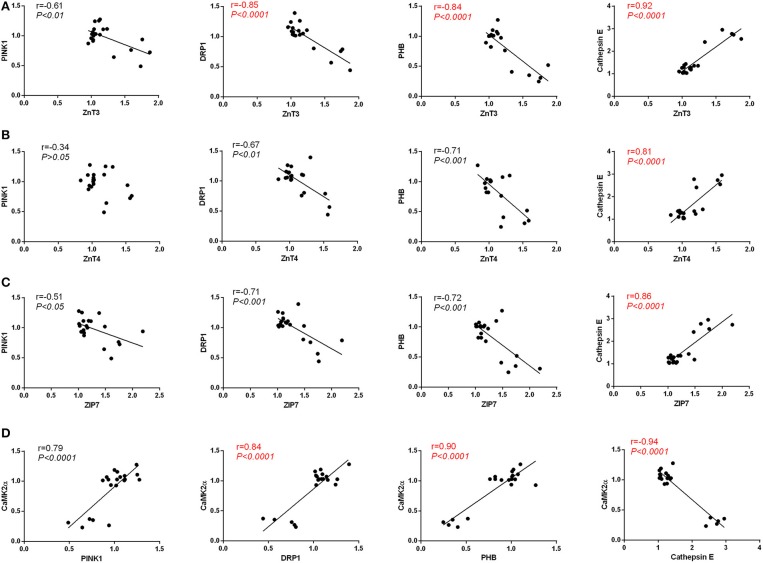
**(A)** Pearson's correlation between the protein level of ZnT3 and the levels of PHB, Pink1, DRP1, and Cathepsin E in hippocampus. **(B)** Pearson's correlation between the protein level of ZnT4 and the levels of PHB, Pink1, DRP1, and Cathepsin E in hippocampus. **(C)** Pearson's correlation between the protein level of ZIP7 and the levels of PHB, Pink1, DRP1, and Cathepsin E in hippocampus. **(D)** Pearson's correlation between the protein level of CaMKIIα and the levels of PHB, Pink1, DRP1, and Cathepsin E in hippocampus.

As is shown in Figure 9A, we found a strong negative correlation between the protein level of ZnT3 and the level of Pink1(Pearson's *r* = −0.61, *P* < 0.01), DRP1(Pearson's *r* = −0.85, *P* < 0.0001) and PHB (Pearson's *r* = −0.84, *P* < 0.0001), and a strong positive correlation between the protein level of ZnT3 and Cathepsin E(Pearson's *r* = 0.92, *P* < 0.0001); in Figure 9B, we found a strong negative correlation between the protein level of ZnT4 and the level of DRP1(Pearson's *r* = −0.67, *P* < 0.01) and PHB (Pearson's *r* = −0.71, *P* < 0.001), and a strong positive correlation between the protein level of ZnT4 and Cathepsin E(Pearson's *r* = 0.81, *P* < 0.0001), whereas, none of such relationship was found in the protein level of ZnT4 and Pink1(Pearson's *r* = −0.34, *P* > 0.05); in Figure 9C, we found a strong negative correlation between the protein level of ZIP7 and the level of Pink1(Pearson's *r* = −0.51, *P* < 0.05), DRP1(Pearson's *r* = −0.71, *P* < 0.001) and PHB (Pearson's *r* = −0.72, *P* < 0.001), and a strong positive correlation between the protein level of ZIP7 and Cathepsin E(Pearson's *r* = 0.86, *P* < 0.0001); in Figure 9D, We observed a strong positive correlation between the protein level of CaMKII_α_ and the levels of PHB(Pearson's *r* = 0.90, *P* < 0.0001), Pink1(Pearson's *r* = 0.79, *P* < 0.0001) and DRP1(Pearson's *r* = 0.84, *P* < 0.0001) and a strong negative correlation between the protein level of CaMKII_α_ and Cathepsin E (Pearson's *r* = −0.94, *P* < 0.0001) in hippocampus.

### Co-immunoprecipitation (co-IP)

**Figure 10:**
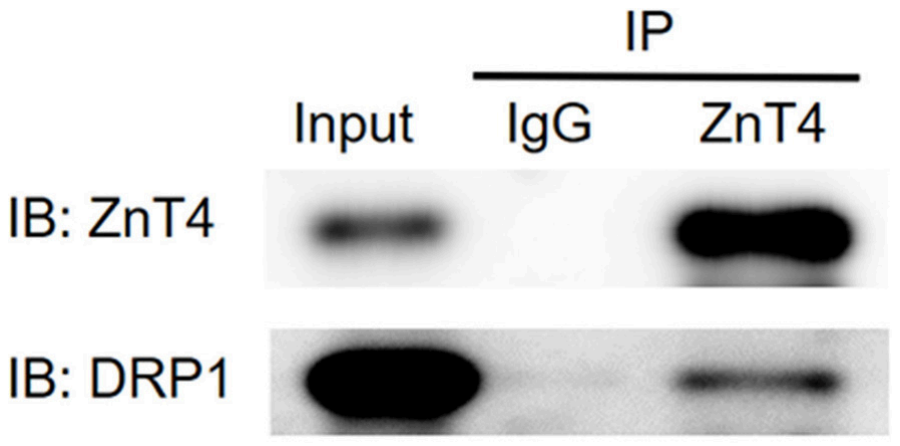
Interaction of ZnT4 and DRP1 was examined by co-immunoprecipitation (co-IP). IP: represent Co-immunoprecipitation (co-IP) assay was performed with goat IgG (negative control) and goat anti-ZnT4 antibody; IB: represent western blotting procedure with goat anti- ZnT4 and mouse anti-DRP1 antibodies after IP; Input: represent positive control (supernatants of hippocampus Extracts).

**C**o-IP assay was performed to check the interaction of ZnT4 with DRP1 protein. As shown in Figure 10, DRP1 protein was detected in immunoprecipitation obtained with anti-ZnT4 antibody. This indicated that ZnT4 interacted with DRP1 in hippocampus.

## Discussion

In the present study, it was found that continuous (10 days) leptin treatment counteracted the long-term increased sprouting of hippocampal mossy fibers and cognitive impairment, as well as corrected the abnormal expressions of related gene in the hippocampus, including mitochondria markers PHB, PINK1, DRP1, and its executive molecular Cathepsin E, zinc transporter 3,4 (ZnT3, ZnT4), ZIP7 and memory marker CaMK II α. These findings suggest that zinc/CaMK II-associated mitophagy signaling may be involved in the long-term neurobehavioral and neuropathological changes caused by neonatal seizures, thus providing new clues to translational medicine in search of potential targets for epileptogenesis.

Leptin has recently been shown to slow down neuronal damage following acute brain injury and long-term neurodegeneration, suggesting that leptin has neurotrophic and neuroprotective activities (26). Platt et al. reported that leptin-resistant mice had elevated levels of tau phosphorylation, while cells cultured with leptin reduced tau phosphorylation (27). Using traumatic brain injury (TBI) mouse models, Lopez Rodriguez et al. demonstrated that leptin (once, i.p. immediately after lesion) restored several parameters of TBI including the expression of cannabinoid receptors, axonal damage, and neuroinflammatory components (28). Malekizadeh et al further demonstrated that leptin fragments promote activity-dependent hippocampal synaptic plasticity, enhance cognitive function, and prevent hippocampal synaptic destruction and neuronal cell death (29). On the other hand, little is known about the protective effect of leptin on convulsive brain injury, especially for the developmental brain. Jayaram et al reported that leptin receptor knockout in mice resulted in a higher lethality in the early stage of seizures and that leptin pretreatment could attenuate glial cytotoxicity induced by excess glutamate *in vitro* (10). Obeid et al. studied the acute and long-term neuroprotective effects of leptin on kainic acid induced status epilepticus (SE) in adult Sprague-Dawley rats. They found that leptin reduced cell damage 24 h after KA-induced SE. However, leptin failed to prevent long-term behavioral defects at 6 weeks after SE (11). However, we found in this study that administration of leptin for 10 consecutive days immediately after neonatal seizure significantly reduced the long-term hippocampal pathological damage and cognitive impairmen. We speculate that the dose of leptin may be the leading cause of the differences with the Obeid's studies described above. In Obeid's study, three doses of leptin (1 h 4 mg/kg, 13 and 24 h 2 mg/kg) were injected intraperitoneally after KA (KALEP group) for a total dose of 8 mg/kg. However, in our study, the dose for 10 consecutive days was 2 mg/kg/day and the total dose was 20 mg/kg. Other factors, such as the model of seizure and the timing of leptin therapy, may also affect outcomes. In this study, a neonatal convulsive model was used and the BBB was still in development and its permeability to leptin may be higher than in adult rats. It is noteworthy that our results are partly in line with the recently published study by Mela et al. They investigated the effects of maternal deprivation (MD) -induced long-term hippocampal and cortical changes in rats and leptin treatment in neonates. The results showed that leptin treatment (3 mg/kg/day, sc, P9-P13) partially counteracted the neurobehavioral changes in open-field tests in neonatal MD rats (30). To the best of our knowledge, the impact of chronic leptin treatment on epileptic-induced brain damage has never been evaluated. In this study, we identified leptin as a neuroprotective effect on neonatal convulsive brain injury. In addition, we explored the possible molecular signaling pathways of leptin protection.

Previous studies have shown that elevated expression of lipid metabolism-related proteins such as ApoE, clusterin, and cholesterol in AD pathology is associated with the zinc metabolic signaling (31, 32). Our previous study also found that chronic ketogenic diet treatment modulated the long-term expression of ZnT-3, MT-3, ApoE/ApoJ, and the autophagy markers Beclin-1, LC3, p62, and Cathepsin-E in hippocampus after neonatal seizures (19). Therefore, we speculate that zinc/lipid metabolism-associated autophagy pathway may be potential targets for the treatment of brain injury caused by epilepsy. The current results provide evidence to support this assumption. Here, compared with the control, non-leptin treated RS group showed significant increase on the protein levels of ZnT3, ZnT4, ZIP7 and Cathepsin-E, as well as down-regulated mitophagy markers PHB, PINK1, DRP1 in hippocampus, linear correlation analysis exhibited further significant correlations between PHB-ZnT3, PINK1-ZnT3, DRP1-ZnT3, CathepsinE-ZnT4, and CathepsinE-ZIP7 which was in parallel with hippocampal mossy fiber sprouting and cognitive abnormalities as previously reported (23, 33). The results are consistent with our recent findings that a significantly up-regulation of ZnT3 and the autophagy markers of Beclin-1, p62 and Cathepsin-E was induced by recurrent neonatal seizure in the hippocampus which was counteracted by leptin or chronic ketogenic diet treatment (20). Other evidence supporting the neuroprotective effects of leptin through autophagy signaling is derived from the finding that leptin therapy reduced cerebral ischemic injury by inhibiting the elevation of connexin 43 *in vivo* (34). Connexins have recently emerged as substrates and regulators of autophagy. Several connexin isoforms could regulate autophagy by recruiting pre-autophagosomal autophagy-related proteins to the plasma membrane (35). For instance, 6 h after traumatic brain injury (TBI), p-CX43 and LC3-II expression reached a maximum level in the hippocampus of rats, while the inhibition of p-CX43 reduced the TBI-induced autophagy. This suggested a regulatory role of connexin 43 in autophagy signaling in the hippocampal neurons following TBI (28). Increased autophagy activity of osteocyte-like MLO-Y4 cells induced the internalization of connexin 43 into autophagosome/autolysosomes and was degraded by autophagy (36). Taken together, these findings, combined with our current findings, in particular the co-precipitation of ZnT4 with the mitochondrial autophagy marker DRP1, underscore the hypothesis that zinc metabolism-related mitophagy signaling may serve as a new target for leptin therapy for developmental convulsive brain injury.

Notably, we here also found that the expression level of CaMKIIα in neonatal was very low and its expression tendency was the same as that of the mitophagy markers (PHB, PINK1, DRP1) and in contrast to Cathepsin-E. Linear correlation analysis further exhibited significant correlations between PHB-CaMKIIα, DRP1-CaMKIIα and CathepsinE-CaMKIIα. The results are quite in line with Bigford's report. They found a recruitmention of pCaMKII to the membrane rafts and a translocation of Beclin-1 export membrane microdomains was rapidly induced by moderate traumatic brain injury (37). It has been documented that CaMKII signaling regulates apoptosis and autophagy simultaneously in cancer cells and in liver in adaptation to starvation (38). In addition, our present results also showed significant negative linear correlations between ZnT3-CaMKIIα, ZnT4-CaMKIIα, and ZIP7-CaMKIIα. This is in accordance with previous findings that zinc inhibited Ca2+/calmodulin-stimulated autophosphorylation and substrate phosphorylation activity in rat cerebral cortex (39). Based on these data, the interaction between CaMKIIα, mitophagy and zinc transporter signaling may be related to neonatal seizure-induced hippocampal mossy fiber sprouting and cognitive impairment as well, which merits further investigations.

## Conclusion

In summary, this study suggests that a zinc/CaMK II related mitophagy signaling is associated with neonatal seizure-induced long-term brain damage in the aspects of hippocampal pathological and cognitive deficits. Moreover, it is effective for chronic leptin treatment to counteract these neuroanatomical, cognitive and molecules changes, suggesting a potential clinical significance.

This study has some limitations. If we perform immunohistochemical protein analysis and Western blot analysis of hippocampus in different regions, it would be more conducive to reveal the intrinsic correlation between Timm staining results and Western blotted protein data.

## Ethics statement

This study was carried out in accordance with the recommendations of the NIH Guide for the Care and Use of Laboratory Animals and approved by the Institutional Animal Care and Use Committee at the Children's Hospital of Soochow University. The protocol was approved by the Medical Ethics Committee of the Children's Hospital of Soochow University.

## Author contributions

HN was the designer and dissertation writer of this study. L-lL was the specific operator of this experiment and was responsible for the statistical analysis of data. M-fJ assisted in the completion of some experiments.

### Conflict of interest statement

The authors declare that the research was conducted in the absence of any commercial or financial relationships that could be construed as a potential conflict of interest.
